# The Relationship between Estimated Glomerular Filtration Rate and Diabetic Retinopathy

**DOI:** 10.1155/2015/326209

**Published:** 2015-03-18

**Authors:** Jingyang Wu, Jin Geng, Limin Liu, Weiping Teng, Lei Liu, Lei Chen

**Affiliations:** ^1^Department of Ophthalmology, The First Hospital of China Medical University, Shenyang 110001, China; ^2^Key Laboratory of Endocrine Diseases of Liaoning Province, The First Hospital of China Medical University, Shenyang 110001, China; ^3^Department of Epidemiology, School of Public Health, China Medical University, Shenyang 110001, China

## Abstract

Diabetic retinopathy (DR) is the leading cause of visual impairment and blindness in working-aged people. Several studies have suggested that glomerular filtration rate (GFR) was correlated with DR. This is a hospital-based study and the aim of it was to examine the relationship between the GFR and DR in patients with type 2 diabetes mellitus (T2DM). We used CKD-EPI equation to estimate GFR and SPSS 19.0 and EmpowerStats software to assess their relationship. Among the 1613 participants (aged 54.75 ± 12.19 years), 550 (34.1%) patients suffered from DR. The multivariate analysis revealed that the risk factors for DR include age (*P* < 0.001, OR = 0.940), duration of diabetes (*P* < 0.001, OR = 1.163), hemoglobin A1c (*P* = 0.007, OR = 1.224), systolic blood pressure (*P* < 0.001, OR = 1.032), diastolic blood pressure (*P* = 0.007, OR = 0.953), high density lipoprotein cholesterol (*P* = 0.024, OR = 3.884), and eGFR (*P* = 0.010, OR = 0.973). Through stratified analysis and saturation effect analysis, our data suggests that eGFR of 99.4 mL/min or lower might imply the early stage of DR in diabetic patients. Thus, the evaluation of eGFR has clinical significance for the early diagnosis of DR.

## 1. Introduction

The prevalence of type 2 diabetes mellitus (T2DM) has significantly increased worldwide in the past 25 years [1, 2]. DR is one of the most common microvascular complications of diabetes mellitus (DM) and is the leading cause of visual impairment and blindness in working-aged people. In recent years, the prevalence of DR is rapidly increasing [3, 4], as the number of people with T2DM increased. The results of a cross-sectional study in a multiethnic Asian population showed that the overall age-standardized prevalence of DR was 25.4% (20%, 24.8%, and 28.9% in Chinese, Malays, and Indians, resp., *P* = 0.290) [5]. More than 50% of T2DM patients likely suffer from DR within twenty years after diagnosis [6]. Because the symptoms of DR are not apparent in early stages of this disease, patients often miss the best opportunity for treatment when diagnosed, leading to a high rate of blindness. According to the World Health Organization (WHO), DR accounts for 4.8% of the total cases of blindness (thirty-seven million worldwide) in 2006 [7]. Therefore, it is important to investigate the risk factors that promote or predict DR.

Diabetic nephropathy (DN), also known as diabetic kidney disease or diabetic glomerulosclerosis, is another major complication of DM and the leading cause of end-stage renal disease (ESRD). GFR and microalbuminuria are clinically significant markers for the evaluation of renal function. Previous studies have shown that microalbuminuria not only is an important clinical marker for DN, but also is closely associated with the progression of DR [8]. However, suffering from DR and the appearance of microalbuminuria do not occur at the same time. GFR describes the flow rate of filtered fluid through the kidney and can be estimated using formulas, thereafter referred to as estimated GFR (eGFR). Unlike microalbuminuria, GFR increases during the early stages of DM due to high blood sugar and decreases during the later stages of DM, reflecting a decline in renal function. That is to say, changes in GFR appear earlier than microalbuminuria in diabetic patients. Past studies have reported that GFR is but one variable of many that affects the likelihood of developing DR and the other complications of DM [9, 10]. In addition, due to the limited medical condition, routine funduscopic examination or microalbuminuria cannot be performed in primary hospital in rural China, especially poverty-stricken areas. So we wondered if eGFR could be used for the early detection of DR, in order to screen it in the general population. If possible, eGFR could be used for screening in the general population.

Hence, our researchers are embarking on a series of studies to investigate the relationship between GFR and DR, and this is the baseline study. The aim of this study was to evaluate the prevalence of DR in hospital-based T2DM patients and investigate the correlation between GFR and DR, so that GFR could be used for DR screening, especially in primary hospital of poor areas.

## 2. Materials and Methods

### 2.1. Study Participants

We conducted a hospital-based case-control study. DM patients were admitted to the Department of Endocrinology and Ophthalmology of the First Affiliated Hospital in China Medical University from September 2010 to March 2012. A total of 1613 patients, 844 (52.3%) males and 769 (47.7%) females, with T2DM were enrolled in this study after rigorous diagnosis and exclusion criteria. The project was performed in accordance with the principles of the Declaration of Helsinki and approved through the Ethics Committee of China Medical University.

### 2.2. Diagnosis and Exclusion Criteria

Diabetes was diagnosed according to the 2006 World Health Organization (WHO) criteria [11]. The DR severity was classified according to the International Clinical Diabetic Retinopathy Disease Severity Scale [12]. Patients were excluded if they had type 1 diabetes mellitus, acute metabolic disorders (such as diabetic ketoacidosis and a hyperglycemic hyperosmolar state), opaque refractive media of one or both eyes (affecting fundus observation), or other eye diseases or serious illnesses (such as cancer). Because DN is often complicated with DR [13], these patients were not excluded to avoid selection bias.

### 2.3. Research Design and Evaluation

The clinical data were extracted from the medical records of 1613 T2DM patients. Information, including the age, gender, duration of diabetes, family history of diabetes, and history of hypertension, was collected for each patient. The blood pressure was measured during the medical examination and recorded as the means of two measurements after the patients rested for 5 minutes. Fasting blood was drawn from the cubital vein and used for biochemical assays, including fasting blood glucose (FBG), glycosylated hemoglobin (HbA1c), triglycerides (TG), total cholesterol (TC), high density lipoprotein cholesterol (HDL-C), low-density lipoprotein cholesterol (LDL-C), serum creatinine (Scr), and blood urea nitrogen (BUN). For the 2-hour postprandial glucose (2hPG) measurement, blood was drawn at 2 hours after ingestion of 75 g glucose powder or bread (equivalent amount of carbohydrates). All samples were measured using the ARCHITECT c8000 biochemical analyzer (Toshiba, Tokyo, Japan). The eGFR was calculated using the CKD-EPI equation [14]. The unit of SCR in the CKD-EPI formula should be “mg/dL,” while the levels of SCR through colorimetric method were in “*μ*mol/L.” Therefore, we converted SCR according to the formula “1 mg/dL = 17.1 *μ*mol/L” and put it into the formula to obtain eGFR.

Direct ophthalmoscopy and fundus photography were conducted after the participants were administered mydriatic eye drops to dilate the pupils. The center of the macula and optic disc of both eyes was photographed using a 45-degree digital camera (CR-DGI, Canon, Japan). Two trained ophthalmologists performed the diagnosis of DR. If there were different opinions, they would discuss to make decision or consult the superior. Based on the results of the assessment, the subjects were divided into two groups: a DR group and a non-DR (DM without DR) group. There were 550 patients in DR group (male 269, female 281) and 1063 patients in NDR group (male 575, female 488).

### 2.4. Statistical Analysis

All statistical analyses were performed using the Statistical Package from the Social Sciences (SPSS) version 19.0, EmpowerStats (http://www.empowerstats.com/), and MedCalc software programs. The basic description and logistic regression analyses were performed using SPSS 19.0 software. The stratified analysis, the interaction test, covariate screening, and curve fitting were performed using EmpowerStats statistical software. MedCalc was used to draw the receiver operating characteristic curve (ROC curve), a graphical plot illustrating the performance of a binary classifier system with varying discrimination thresholds. The area under the ROC curve (AUC) ranged from 0.5 to 1. The numerical variables of normal distribution were expressed as the means ± standard deviation (means ± SD) and percentage (%). The independent samples *t*-test was used to analyze the continuous variables, whereas the odds ratio (OR) and chi-square (*χ* ^2^) test was used to analyze the categorical variables. *P* < 0.05 was considered as statistically significant.

## 3. Results

Based on the exclusion criteria, 1613 T2DM patients aged 54.75 ± 12.19 years were selected into this study. Among these subjects, 550 (34.1%) patients were diagnosed with DR. No differences in TG, DBP, and BUN were detected between the DR and NDR groups. In contrast, statistically significant differences were detected for age (*P* = 0.003), family history of DM (*P* = 0.036), duration of DM (*P* < 0.001), FBG and 2hPG (*P* < 0.001), HbA1c (*P* = 0.003), TC (*P* < 0.001), HDL-C (*P* = 0.013), LDL-C (*P* = 0.002), SBP (*P* < 0.001), DBP (*P* = 0.033), eGFR (*P* < 0.001), and SCR (*P* < 0.001) (Table 1).

To determine the risk factors for DR, a logistic regression model was performed. DR was used as the dependent variable. Different risk factors, identified in the univariable analysis, were used as independent variables (Table 2). There were significant associations between DR and age (*P* < 0.001), DM duration (*P* < 0.001), HbA1c (*P* = 0.007), HDL-C (*P* = 0.024), SBP (*P* < 0.001), DBP (*P* = 0.007), and eGFR (*P* = 0.010). According to the severity of the disease, DR patients were divided into 3 groups: nondiabetic retinopathy (NDR), nonproliferative diabetic retinopathy (NPDR), and proliferative diabetic retinopathy (PDR) groups. The mean value of eGFR was 106.27 ± 23.37, 100.12 ± 30.85, and 83.33 ± 33.77 (mL/min) for the NDR, NPDR, and PDR groups, respectively.

In order to accurately study the relationship between eGFR and DR, we need to excluded the influence factors that have an effect on their relationship through stratified analysis, interaction tests, and covariate screening. First of all, we analyzed the relationship between DR and all risk factors through stratified analysis (Table 3). Each continuous variable was divided into three groups according to its value from low to high. “Sex” group was divided into “male” and “female” groups on the basis of gender. “FHD” group was divided into “No” (not having family history of DM) and “Yes” (having family history of DM) group. The results indicated that there were no confounding factors between the layers. In addition, the relationships between DR and TG and DR and DBP were not identified using univariate analysis but were observed using stratified analysis. Interaction tests were performed to detect the influence of each stratified factor on the relationship between eGFR and DR. *P* ⩽ 0.05 means interaction exists between that factor and the relationship. The presence of effect modifiers of eGFR and DR was observed. Eligible factors, which as effect modifiers for the relationship between eGFR and DR, include SCR (*P* = 0.003), SBP (*P* = 0.043), LDL-C (*P* = 0.025), TC (*P* = 0.005), and BUN (*P* = 0.062) (*P* = 0.062 means there is certain effect modification).

Covariate screening was analyzed using computer software. The screening criteria included risk factors producing >10% change in the regression coefficient after introduction into the basic model. The results showed that the SBP, FBG, 2hPG, HbA1c, and the DM duration met the filter criteria (OR change was 13.5, 13.4, 16.5, 18.5, and 39.0, resp.).

**Figure 1:**
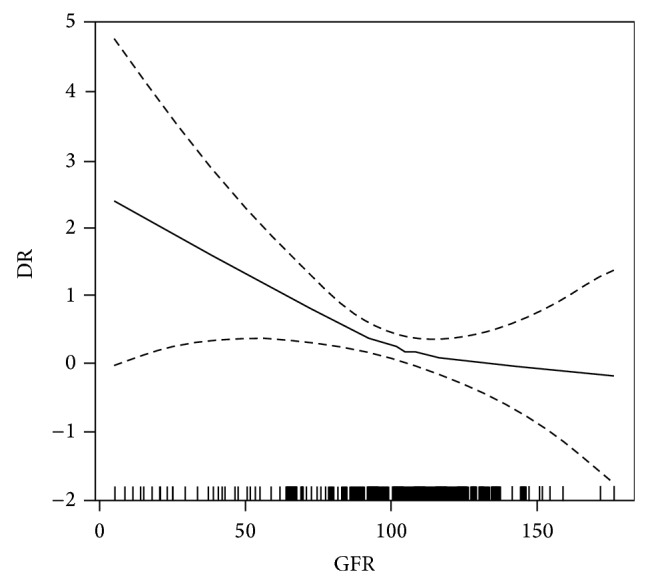
Smooth curve fitting of DR and eGFR. Adjustment variables: duration of DM, FBG, 2hPG, HbA1c, LDL, TC, BUN, SCR, and SBP.

**Figure 2:**
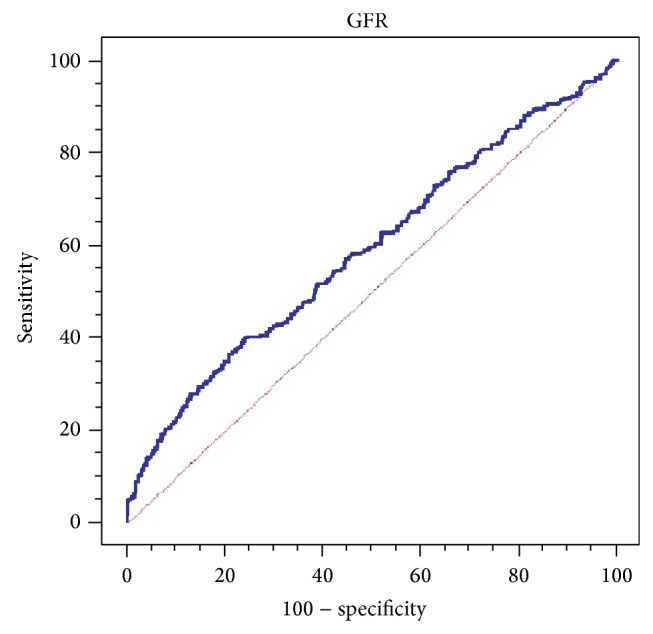
ROC curve of the relationship between DR and eGFR.

After the adjustment of the variables affecting the relationship between eGFR and DR, result of univariate analysis suggested that eGFR remained significantly associated with DR (*P* < 0.001) (Table 4). In addition, smooth curve fitting was performed after the adjustment of all variables, and the resultant curve exhibited a two-stage change and a breakpoint (Figure 1). When the eGFR value was more than the point, the risk of DR was low; however if the value was less than the point, the risk of DR significantly increased. The saturation effects were analyzed based on the curve, and the data indicated that the inflection point was 99.4 mL/min (34th percentile). Before and after the adjustment of the covariates, the logarithmic likelihood ratio test *P* value decreased from 0.050 to 0.036 (Table 5). The results of the ROC curve are shown in Figure 2. The area under the ROC curve (AUC) was 0.591815. Combined with clinical significance, the cut-off value remained 99.4%. The sensitivity and specificity of this point were 42.77% and 70.25%, respectively.

## 4. Discussion

Diabetic retinopathy is one of the most common microvascular complications of DM [15]. With an increase in morbidity of DM, the prevalence of DR has increased yearly, becoming the leading cause of visual impairment and blindness in working-aged people. Thus, it is important to characterize the incidence of DR and identify key factors for predicting it.

Similar to other hospital-based epidemiologic studies in urban China [16], the prevalence of DR in the this study was 34.1%, which is much higher than in Europe, the United States, South Korea, and the other developed countries [17, 18]. This potentially reflects the higher incidence of DM in China [2] and the poor knowledge or less attention to the complications of DM, particularly DR [19]. To reduce the prevalence of DM and slow down the progression of DR, further efforts should be exerted on concerning the public health education of DM and the complications of it.

Many studies have described the risk factors for DR, which primarily include the extended duration of DM, old age, hyperglycemia, hyperlipidemia, proteinuria, severe obesity, alcohol consumption, genetic factors, and the expression of a variety of hormones, such as growth hormone [20–23]. Similarly, the present study confirmed that age, extended duration of DM, high blood sugar, high blood pressure, and hyperlipidemia were significantly associated with DR. In addition, we also showed that eGFR was negatively correlated with DR, consistent with previous studies [13, 24]. These results suggest that measurements of eGFR might help to predict the early stage of DR.

Then we successively conducted stratified analysis, interaction test, and covariate screening to get rid of the factors that affect the relationship between DR and eGFR. After the adjustment of these influence factors, the results still suggested that eGFR was significantly associated with DR. The graphical representation of the relationship between them displayed a two-stage pattern and that the value of eGFR less than or equal to 99.4 mL/min was significantly correlated with DR. The AUC was 0.591815, a value between 0.5 and 0.7, indicating that the diagnostic value was not very useful, likely reflecting a bias in this study based on the fact that absence of the related variables with GFR, like urine protein or microalbumin. In addition, recent studies have shown that the incidence of kidney disease in some diabetic patients was independent of DN, referred to as nondiabetic renal disease (NDRD) [25, 26]. The renal tissue pathological examination revealed that both DN and NDRD reduced GFR levels. DN and DR normally appear in the same person at the same time, so we cannot artificially exclude the patients suffering from DN. However, NDRD patients may consist in the study population and their results could reduce the value of GFR, which affected the results of experimental. Therefore, there may have been selection bias in the study objects.

GFR is an important indicator of kidney function and an important factor in the diagnosis of diabetic nephropathy [27]. Although the direct relationship between GFR and other microangiopathy of DM has not been fully established, high levels of GFR have been negatively associated with the onset of macroangiopathy, such as coronary artery disease [28, 29]. A recent study has also confirmed that DR is closely associated with regional arterial stiffness [30]. Many studies have confirmed that both DR and DN are microvascular complications with similar pathological bases, associated with DM [31–33]. A report from the Atherosclerosis Risk in Communities (ARIC) Study demonstrated that retinopathy and renal dysfunction have a strong association, independent of age, diabetes, hypertension, and other risk factors. Systemic markers of inflammation and endothelial dysfunction associated with retinal vascular abnormalities could contribute to the development of kidney disease [34]. Animal studies have also shown that pathological changes in the retina are highly associated with renal microcirculation [35].

For DM patients, DR and DN have a common pathological basis and a similar course of evolution. Hyperglycemia causes glomerular hyperperfusion and high filtration, leading to an increase of GFR during the early stages of T2DM [36]. The accumulation of advanced glycation end products, due to hyperglycemia, promotes mesangial proliferation and basement membrane thickening in the glomerulus. In addition, the activation of the polyol pathway, the protein kinase C pathway, the pentose phosphate pathway [37], oxidative stress, and various cytokines cause a range of variations in kidney, which include capillary obstruction, a reduction of podocyte proliferation, the loss of the urinary proteins, and a decline in renal function. With further thickening of the glomerular basement membrane, the mesangial matrix increases, resulting in the appearance of cracks and an increase in urinary protein leakage [38]. Accordingly, changes in GFR appear earlier than those in urine protein levels, and these changes remain throughout the entire course of DM.

The pathological mechanisms described above are similar to those observed in the retina. As in the kidney, the high blood sugar exerts deleterious effects on the retina, which include the apoptosis of Muller cells, ganglion cells [39], and pericytes, the thickening of the capillary basement membrane, and the proliferation of endothelial cells in the retina. These effects lead to pathological changes in DR, including nonperfusive capillaries, the appearance of microaneurysms, and exudation. Hence, GFR not only may be an important clinical marker for DN, but also could be correlated with DR.

Taken together, these data highlight the use of eGFR as a predictor of DR. However, the results of the present study are important to the primary hospital for DR screening, especially to the ones with limited resources in China. But there are still some limitations. First, this study was a retrospective case-control study, including hospital-based patients with DM. Second, DR was diagnosed using ophthalmoscopy and fundus photography, but not fundus fluorescein angiography (FFA). Third, the GFR values were estimated by formula, and some of the factors associated with GFR, such as urinary protein excretion or microalbuminuria, were not included in this study. Therefore, a long-term follow-up study should be performed in the future. Moreover, more factors should be included in this study to provide an in-depth examination of the relationship and mechanism of GFR and DR.

In summary, the results of the present study indicate that GFR may have certain implications for DR, which is important to DR screening in primary hospital of China, especially in poverty-stricken areas. Additional studies are needed to explore the mechanisms that couple GFR and DR.

## Figures and Tables

**Table 1:** Clinical characteristics of the participants.

Item	NDR	DR	*P* value
*N* (male/female)	1063 (575/488)	550 (269/281)	
FHD	336 (39.8%)	200 (45.9%)	0.036
Age (year)	54.09 ± 12.71	56.04 ± 11.19	0.003
DD (year)	6.06 ± 5.50	10.38 ± 6.60	<0.001
FBG (mmol/L)	9.15 ± 3.62	10.25 ± 6.43	<0.001
2hPG (mmol/L)	16.91 ± 6.21	18.74 ± 8.62	<0.001
HbA1c (%)	8.26 ± 2.24	8.74 ± 2.16	0.003
TG (mmol/L)	2.25 ± 2.20	2.42 ± 2.57	0.060
TC (mmol/L)	4.89 ± 1.24	5.23 ± 1.56	<0.001
HDL-C (mmol/L)	1.11 ± 0.29	1.16 ± 0.33	0.013
LDL-C (mmol/L)	3.11 ± 1.00	3.35 ± 1.20	0.002
SBP (mmHg)	132.29 ± 21.00	139.16 ± 23.94	<0.001
DBP (mmHg)	82.75 ± 11.32	83.99 ± 11.76	0.033
eGFR (mL/min)	106.25 ± 23.32	95.27 ± 31.98	<0.001
SCR (*μ*mmol/L)	65.16 ± 36.72	83.01 ± 82.61	<0.001
BUN (mmol/L)	6.61 ± 7.87	7.28 ± 5.04	0.062

NDR: nondiabetic retinopathy; DR: diabetic retinopathy; FHD: family history of diabetes mellitus; DD: duration of diabetes mellitus; FBG: fasting blood glucose; 2hPG: 2-hour postprandial blood glucose; HbA1c: hemoglobin A1c; TG: triglyceride; TC: total cholesterol; HDL-C: high density lipoprotein-cholesterol; LDL-C: low density lipoprotein-cholesterol; SBP: systolic blood pressure; DBP: diastolic blood pressure; GFR: glomerular filtration rate; Scr: serum creatinine; BUN: blood urea nitrogen.

**Table 2:** The logistic regression analysis of the risk factors for DR.

Variables	DR odds ratio (95% CI)	*P* value	*β*
Sex	0.933 (0.545–1.599)	0.802	
FHD	1.186 (0.693–2.029)	0.534	
Age (year)	0.940 (0.911–0.971)	<0.001^*^	−0.062
DD (year)	1.163 (1.107–1.222)	<0.001^*^	0.151
FBG (mmol/L)	0.979 (0.885–1.082)	0.676	
2hPG (mmol/L)	1.027 (0.971–1.085)	0.351	
HbA1c (%)	1.224 (1.056–1.418)	0.007^*^	0.202
TG (mmol/L)	1.094 (0.828–1.447)	0.526	
TC (mmol/L)	0.685 (0.322–1.460)	0.327	
HDL-C (mmol/L)	3.884 (1.191–12.672)	0.024^*^	1.357
LDL-C (mmol/L)	1.172 (0.547–2.513)	0.620	
SBP (mmHg)	1.032 (1.015–1.050)	<0.001^*^	0.032
DBP (mmHg)	0.953 (0.920–0.987)	0.007^*^	−0.048
eGFR (mL/min)	0.973 (0.955–0.991)	0.010^*^	−0.023
SCR (*μ*mmol/L)	1.002 (0.995–1.009)	0.492	
BUN (mmol/L)	0.931 (0.848–1.002)	0.133	

^*^ *P* < 0.05.

**Table 3:** Stratified analysis of all variables and interaction tests.

	*N* (%)	DR	*P* value of interaction
Sex			0.453
Male	518 (53.7%)	0.984 (0.977, 0.991) <0.001	
Female	446 (46.3%)	0.988 (0.981, 0.995) 0.001	
FHD			0.839
No	427 (57.1%)	0.984 (0.977, 0.991) <0.001	
Yes	321 (42.9%)	0.983 (0.974, 0.992) <0.001	
BUN			0.062
Low	317 (33.0%)	0.986 (0.972, 0.999) 0.039	
Medium	324 (33.7%)	1.003 (0.989, 1.018) 0.654	
High	321 (33.4%)	0.984 (0.977, 0.991) <0.001	
SCR			0.030^*^
Low	300 (31.1%)	1.002 (0.987, 1.018) 0.802	
Medium	338 (35.1%)	0.979 (0.964, 0.995) 0.008	
High	326 (33.8%)	0.979 (0.971, 0.986) <0.001	
DBP			0.411
Low	169 (25.9%)	0.980 (0.968, 0.992) <0.001	
Medium	242 (37.1%)	0.990 (0.980, 0.999) 0.036	
High	242 (37.1%)	0.983 (0.974, 0.993) <0.001	
SBP			0.043^*^
Low	138 (21.1%)	0.983 (0.968, 0.998) 0.024	
Medium	232 (35.5%)	0.999 (0.987, 1.010) 0.813	
High	284 (43.4%)	0.981 (0.973, 0.990) <0.001	
LDL			0.025^*^
Low	296 (34.1%)	0.992 (0.983, 1.001) 0.068	
Medium	284 (32.7%)	0.988 (0.978, 0.999) 0.029	
High	289 (33.3%)	0.975 (0.966, 0.985) <0.001	
HDL			0.618
Low	291 (33.5%)	0.986 (0.977, 0.994) 0.001	
Medium	289 (33.3%)	0.981 (0.971, 0.991) <0.001	
High	288 (33.2%)	0.987 (0.978, 0.996) 0.006	
TC			0.005^*^
Low	297 (34.2%)	0.996 (0.987, 1.005) 0.377	
Medium	289 (33.3%)	0.982 (0.972, 0.993) <0.001	
High	283 (32.6%)	0.976 (0.966, 0.985) <0.001	
TG			0.526
Low	294 (33.8%)	0.989 (0.980, 0.998) 0.018	
Medium	290 (33.3%)	0.985 (0.976, 0.994) 0.001	
High	286 (32.9%)	0.981 (0.972, 0.991) <0.001	
HbA1c			0.865
Low	237 (31.7%)	0.980 (0.970, 0.990) <0.001	
Medium	252 (33.7%)	0.984 (0.972, 0.995) 0.004	
High	259 (34.6%)	0.983 (0.971, 0.9958) 0.005	
PBG			0.247
Low	211 (28.7%)	0.976 (0.965, 0.986) <0.001	
Medium	252 (34.2%)	0.983 (0.973, 0.993) 0.001	
High	273 (37.1%)	0.988 (0.978, 0.999) 0.027	
FBG			0.206
Low	284 (31.3%)	0.978 (0.970, 0.987) <0.001	
Medium	297 (32.8%)	0.991 (0.9808, 1.002) 0.093	
High	325 (35.9%)	0.981 (0.971, 0.991) <0.001	
DD			0.213
Low	255 (31.9%)	0.995 (0.982, 1.008) 0.443	
Medium	246 (30.8%)	0.995 (0.984, 1.006) 0.360	
High	298 (37.3%)	0.984 (0.976, 0.993) <0.001	
AGE			0.807
Low	317 (32.9%)	0.985 (0.975, 0.996) 0.005	
Medium	318 (33.0%)	0.985 (0.976, 0.995) 0.003	
High	329 (34.1%)	0.981 (0.972, 0.990) <0.001	

^*^ *P* < 0.05.

Each continuous variable was divided into three groups according to its value from low to high; “Sex” group was divided into male and female; “FHD” group was divided into No (not having family history of DM) and Yes (having family history of DM).

**Table 4:** Correlation analysis after adjustment of the effect modifier.

	Statistics	DR
GFR	102.45 ± 27.09	0.971 (0.954, 0.988) <0.001

Adjustment variables: duration of DM, FBG, 2hPG, HbA1c, LDL, TC, BUN, SCR, and SBP.

**Table 5:** Saturation effect analysis before and after adjustment of the effect modifier.

		Before adjustment	After adjustment
Model I	OR value	0.985 (0.980, 0.990) <0.001	0.988 (0.974, 1.002) 0.089

Model II	Breakpoint (K)	97.330 (31st percentile)	99.400 (34th percentile)
	OR1 (<99.400)	0.979 (0.971, 0.987) <0.001	0.965 (0.939, 0.991) 0.008
	OR2 (>99.400)	0.995 (0.984, 1.006) 0.351	1.003 (0.983, 1.023) 0.779
	OR2/OR1	1.016 (1.00, 1.032) 0.050	1.040 (1.002, 1.078) 0.037
	Logarithmic likelihood ratio test *P* value	0.050	0.036

Adjustment variables: duration of DM, FBG, 2hPG, HbA1c, LDL, TC, BUN, SCR, and SBP.
